# Open source model for generating RR intervals in atrial fibrillation and beyond

**DOI:** 10.1186/1475-925X-6-9

**Published:** 2007-03-02

**Authors:** Jie Lian, Gari D Clifford, Dirk Müssig, Volker Lang

**Affiliations:** 1 Micro Systems Engineering, Inc., 6024 SW Jean Rd., Lake Oswego, OR 97035, USA; 2 Harvard-MIT Division of Health Sciences & Technology, 77 Massachusetts Avenue, Cambridge, MA 02139, USA

## Abstract

**Background:**

Realistic modeling of cardiac inter-beat (RR) intervals is highly desirable for basic research in cardiac electrophysiology, clinical management of heart diseases, and developing signal processing tools for ECG analysis.

**Methods:**

We present an open source computer model that is capable to generate realistic time series of RR intervals in both physiologic and pathologic conditions. Detailed model structure and the software implementation are described.

**Results:**

Examples are provided on how to use this model to generate RR intervals in atrial fibrillation with ventricular pacing, normal sinus rhythm with heart rate variability, and typical atrial flutter with atrioventricular block. The extensibility of the model is also discussed.

**Conclusion:**

The present computer model provides a unified platform wherein various types of ventricular rhythm can be simulated. The availability of this open source model promises to support and stimulate future studies.

## Background

The variation of cardiac inter-beat (PP, RR or NN) intervals results from both rhythmic activity of the heart electrical source and the dynamic properties of the cardiac conduction pathway, both of which are under autonomic control. In normal sinus rhythm, the RR intervals are known to fluctuate at various time scales, a phenomenon known as heart rate variability, which has been extensively investigated to probe the autonomic nervous activity [1]. On the other hand, the abnormal cardiac rhythm, for example during atrial fibrillation (AF), has been thought to mainly result from disturbance in autonomic modulations of the electrophysiological properties of the atria and the atrioventricular (AV) node [2]. Hence, analysis of RR intervals may offer valuable insights into the mechanisms of arrhythmia genesis, maintenance, and termination.

From the therapeutic perspective, characterization of the RR intervals can guide the development of novel strategies for cardiac rhythm management. For instance, many antiarrhythmic drugs are known to affect ventricular rhythm by modifying various electrical properties of the heart, including the automaticity, the conduction velocity, and the refractory period [3]. In another example, specially designed pacing protocols could be used for prevention, termination, or rate regularization of AF through implantation of artificial pacemakers [4].

Consequently, time series analysis of RR intervals has been a research thrust in the field of biomedical engineering. Numerous techniques based on RR interval analysis have been developed in the past decades to assess the autonomic control of the cardiovascular system, to assist clinical diagnosis of cardiac disorders, to facilitate arrhythmia predication and risk stratification, and so on [5]. However, quantitative comparison of different analytic methods has been hindered by the heterogeneity of various data sources and the inherent noise and uncontrollability of real world recordings. Therefore, realistic modeling of RR intervals is highly desired to provide a unified platform wherein various algorithms can be evaluated.

Despite the apparent scientific merits and clinical significance, there is a stark paucity of computer models to generate realistic time series of RR intervals. Although several RR interval generators had been created in response to the PhysioNet/Computers in Cardiology Challenge 2002 [6], all these models are limited to synthesizing sinus rhythm with simulated heart rate variability, yet not applicable to abnormal rhythms.

**Figure 1:**
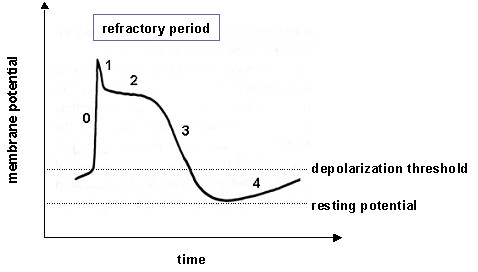
Illustration of the action potential of the AV nodal cells.

Recently, a novel computer model, known as AF-VP, was developed by the authors to elucidate the effects of ventricular pacing (VP) on the ventricular rhythm in AF [7]. The kernel component of the model is the AV junction (AVJ), which is treated as a lumped structure with defined electrical properties mimicking those of individual AV nodal cells. As illustrated in Figure 1, the action potential of the AV nodal cells has five phases. The cell is depolarized (Phase 0) when its membrane potential crosses the depolarization threshold. Then the cell repolarizes (Phases 1–3) and returns to the resting potential (Phase 4). The refractory period, when no new action potential can be initiated, begins with Phase 0 and extends into Phase 3. The AV nodal cells can depolarize spontaneously due to gradual increase of the membrane potential in Phase 4. Such automaticity is usually suppressed by the higher firing rate of the sinus node in atrium, but may manifest in abnormal conditions, such as during sinus node dysfunction or the AV junctional tachycardia.

It has been demonstrated that this AF-VP model could account for most known experimental observations in AF [7]. Of particular note is the plasticity of the model, that is, this model can be easily extended beyond AF, to simulate various types of RR intervals in both normal physiologic and pathologic conditions. In order to facilitate the use and further improvement of the AF-VP model, its software (written in ANSI/ISO C [see Additional file 1]) has been made freely available on PhysioNet [8], an on-line forum for the dissemination and exchange of recorded and simulated biomedical signals and archives of open source software [9].

In this paper, we describe the software architecture of the AF-VP model. Examples are given on how to use this model to synthesize RR intervals for different rhythms, and the extensibility of the model is discussed.

## Methods

### Model structure

**Figure 2:**
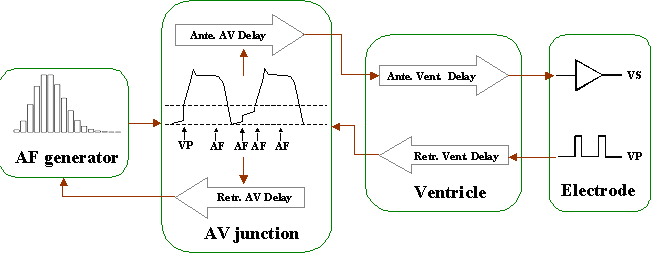
Schematic drawing of the AF-VP model.

Figure 2 illustrates how the AF-VP model is composed of four inter-connected components: the AF generator, the AV junction (AVJ), the ventricle, and the electrode used to record the electrical activity [7].

The AF generator outputs random AF impulses bombarding the AVJ. However, retrograde conducted waves escaping the AVJ can collide with an imminent AF impulse or reset the timing cycle of the AF generator.

The AVJ fires when its membrane potential (V_m_) reaches the depolarization threshold (V_T_). The activation of AVJ starts a refractory period, when the AVJ is refractory to any stimulation. At the end of refractory period, V_m _returns to the resting potential (V_R_) and starts to rise in a linear manner. Each time an AF impulse hits the AVJ during Phase 4, V_m _is increased by a discrete amount ΔV [10,11]. However, if the AVJ is invaded by a VP-induced retrograde wave during Phase 4, V_m _is brought to V_T _directly.

The firing of the AVJ generates an activation wave that starts an antegrade or retrograde AV delay (AVD), depending on the direction of activation. If the AVJ is retrograde activated while an antegrade wave has not finished its AVD or vice versa, a collision occurs, annihilating the activation waves in both directions. Both refractory period and conduction delay of the AVJ are dependent upon its recovery time, which is defined as the interval between the end of last AVJ refractory period and the current AVJ activation time. In addition, the electrotonic modulation of the AVJ refractory period by blocked impulses is also incorporated in the model [7].

After finishing the antegrade AVD, an activation wave is generated in the ventricle and starts the antegrade ventricular conduction. The delivery of VP also generates a retrograde activation wave in the ventricle toward the AVJ. When both antegrade and retrograde waves are present in the ventricle, a ventricular fusion beat manifests, causing the extinction of both waves.

The electrode for recording this activity is assumed to be implanted in the ventricle and connected to a pacing device operating in demand mode. If an activation wave propagates to the electrode after an antegrade ventricular conduction delay, a ventricular sense (VS) occurs that inhibits the scheduled VP, whereas the timeout of the pacing interval triggers the VP.

### Software implementation

**Figure 3:**
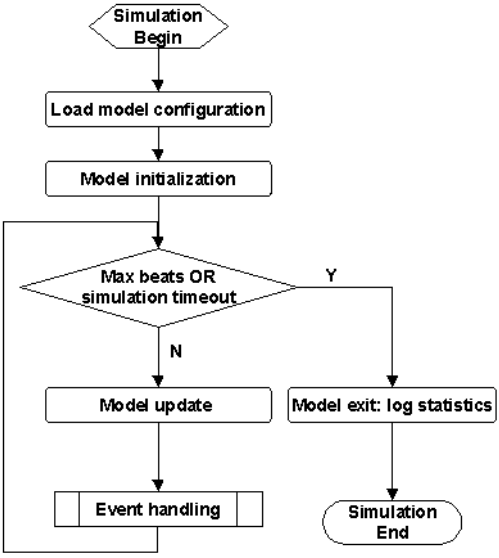
Top-level flowchart of the AF-VP model.

Figure 3 shows the top-level flowchart of the simulation model. The software loads the model parameters from an external configuration file (see Appendix A for an example), and then initializes variables that include various timers and counters as being summarized in Tables 1, 2, respectively. The simulation runs at the programmed sampling frequency (with a default of 1000 Hz). At each sampling time, the model adjusts the timers and updates V_m _(in a linearly increasing manner) if the AVJ is in Phase 4, and then handles possible event(s) as detailed below. The simulation continues until the desired number of cardiac beats (RR intervals) is generated or the simulation time runs out, when the model logs statistics and exits.

**Table 1:** List of timers used in the AF-VP model

Timer	Start condition	Stop condition
AF interval timer	Arrival of AF impulse	End of AF interval
RR interval timer	Any VS or VP event	Next VS or VP event
AVJ refractory timer	AVJ activation	End of AVJ refractory period
VP clock timer	Any VS or VP event	End of VP interval
Antegrade AVJ timer	Antegrade AVJ activation	End of antegrade AVD
Retrograde AVJ timer	Retrograde AVJ activation	End of retrograde AVD
Antegrade ventricle timer	End of antegrade AVD	VS event or ventricle fusion
Retrograde ventricle timer	Delivery of VP	Retrograde invasion of AVJ or ventricle fusion

**Table 2:** List of counters used in the AF-VP model

Counter	Increment condition
AF impulse counter	Arrival of AF impulse
Atrial invasion counter	Retrograde wave escapes the AVJ
AV block counter	Antegrade or retrograde AV block (w/o fusion)
AVJ fusion counter	Collision of antegrade & retrograde waves in AVJ
Ventricular fusion counter	Collision of antegrade & retrograde waves in ventricle
VS counter	VS event
VP counter	VP event
Beat counter	VS or VP event

**Figure 4:**
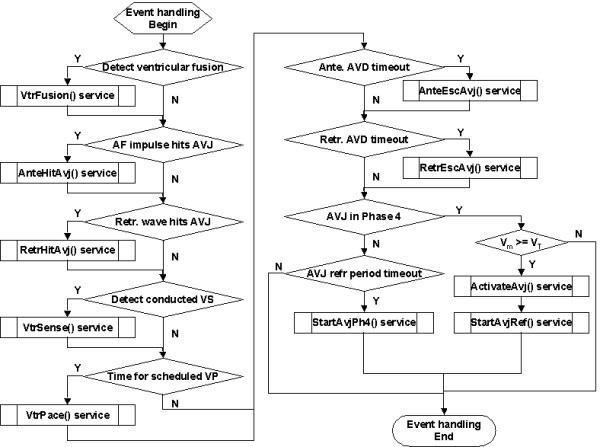
Flowchart of the AF-VP model event handling routine.

The event handling routine is described in Figure 4. Sequentially, the model checks its timers to detect the following events and calls for respective services if any:

- ventricular fusion (*VtrFusion*),

- AF bombardment of AVJ (*AnteHitAvj*),

- retrograde invasion of AVJ (*RetrHitAvj*),

- ventricular sense (*VtrSense*),

- ventricular pace (*VtrPace*),

- antegrade AVD timeout (*AnteEscAvj*), and

- retrograde AVD timeout (*RetrEscAvj*).

The model then checks the status of the AVJ. If the AVJ is in Phase 4 and V_m _≥ V_T_, then services are called for AVJ activation (*ActivateAvj*) and the initiation of the refractory period (*StartAvjRef*). On the other hand, if the AVJ is in refractory period, then no action is taken until its timeout, when a service is called to start the Phase 4 (*StartAvjPh4*).

**Figure 5:**
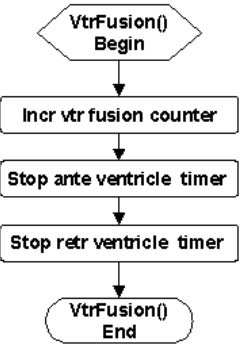
Flowchart of the *VtrFusion *service called upon ventricular fusion.

Ventricular fusion is detected when two opposite traveling waves in the ventricle meet, based on the ratios of active ventricle timers to the fixed ventricular conduction delays in both directions. The *VtrFusion *service (Figure 5) increases the ventricular fusion counter, and then stops both antegrade and retrograde ventricle timers.

**Figure 6:**
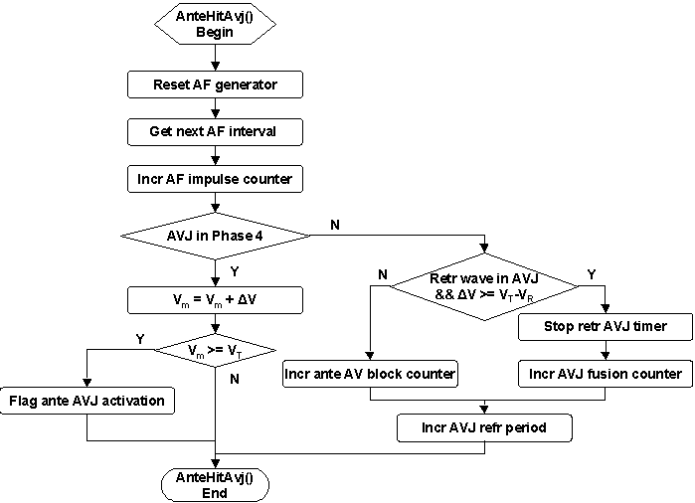
Flowchart of the *AnteHitAvj *service called upon antegrade AF bombardment of the AVJ.

The AVJ is bombarded by an AF impulse at the end of an AF interval. The *AnteHitAvj *service (Figure 6) increments the AF counter, resets the AF generator and obtains the next AF interval. If the AVJ is in Phase 4, then V_m _has a step increase of ΔV. Antegrade AVJ activation is flagged if V_m _≥ V_T_. On the other hand, if the AF impulse hits the AVJ during refractory period, then the AVJ refractory period is extended, simulating the electrotonic modulation effect of the concealed conduction. If there is a retrograde wave in the AVJ and the AF impulse has supra-threshold strength, then the retrograde AVJ timer is disabled (prevent retrograde conduction to atrium) and the AVJ fusion counter is incremented. Otherwise, an antegrade AV block is registered.

**Figure 7:**
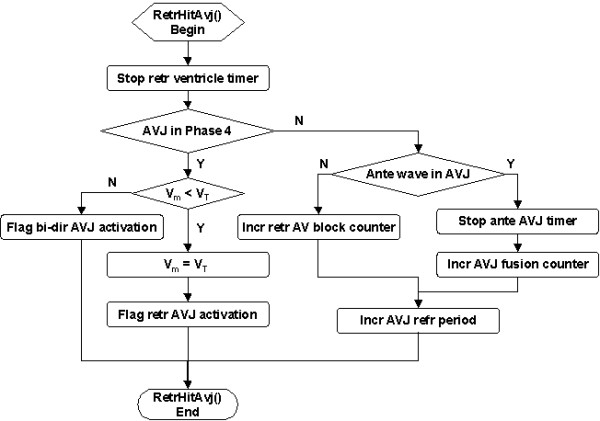
Flowchart of the *RetrHitAvj *service called upon VP-induced retrograde wave invasion of the AVJ.

The AVJ is invaded by a VP-induced retrograde wave when its timer completes the retrograde ventricular conduction delay. The *RetrHitAvj *service (Figure 7) first stops the retrograde ventricular timer. If the AVJ is in Phase 4 and V_m _< V_T_, then V_m _is brought to V_T _directly and the retrograde AVJ activation is flagged. If the AVJ is in Phase 4 but V_m _≥ V_T_, then it implies a rare condition that the AVJ is simultaneously activated from both directions. On the other hand, if the AVJ is in refractory period, then the AVJ refractory period is extended (electrotonic modulation effect). If there is an active antegrade wave in the AVJ, then the antegrade AVJ timer is disabled (prevent antegrade conduction to ventricle) and the AVJ fusion counter is incremented. Otherwise, a retrograde AV block is registered.

**Figure 8:**
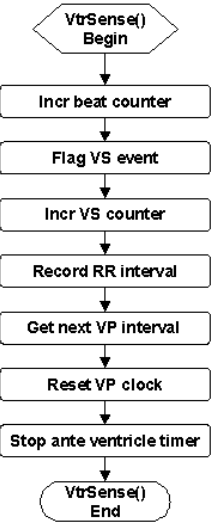
Flowchart of the *VtrSense *service called upon ventricular sense.

A ventricular sense is registered when an antegrade ventricle timer completes the antegrade ventricular conduction delay. The *VtrSense *service (Figure 8) takes a series of steps to flag the VS event, increment the beat counter and the VS counter, record the RR interval, obtain the next VP interval, reset the VP clock (i.e., inhibit VP), and stop the antegrade ventricle timer.

**Figure 9:**
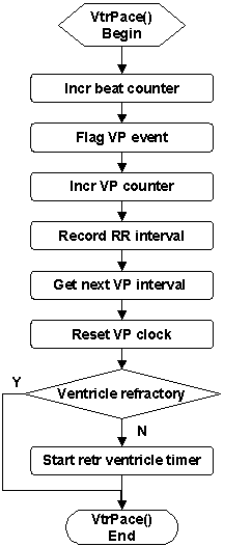
Flowchart of the *VtrPace *service called upon ventricular pace.

A ventricular pace is registered when the VP clock exceeds the VP interval. In parallel to the VS event, the *VtrPace *service (Figure 9) flags the VP event, increments the beat counter and the VP counter, records the RR interval, obtains the next VP interval, resets the VP clock, and starts a retrograde ventricle timer for retrograde conduction if the ventricle is non-refractory (sufficient delay since last excitation).

**Figure 10:**
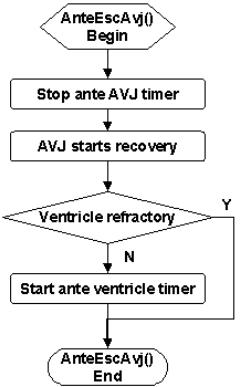
Flowchart of the *AnteEscAvj *service called upon antegrade wave completing AV delay and escaping the AVJ.

An antegrade wave escapes the AVJ after finishing the antegrade AVD. The *AnteEscAvj *service (Figure 10) stops the antegrade AVJ timer, enables the AVJ for recovery, and starts an antegrade ventricle timer for antegrade conduction if the ventricle is non-refractory (sufficient delay since last excitation).

**Figure 11:**
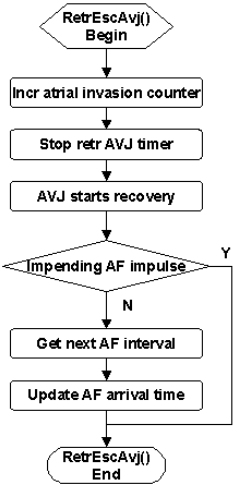
Flowchart of the *RetrEscAvj *service called upon retrograde wave completing AV delay and escaping the AVJ.

Likewise, a retrograde wave escapes the AVJ after completing the retrograde AVD. The *RetrEscAvj *service (Figure 11) increments the atrial invasion counter, stops the retrograde AVJ timer, and enables AVJ recovery. If there is an impending AF impulse, then an atrial collision is expected and there is no impact on next AF arrival time. Otherwise, the AF generator is reset and the next AF interval is updated.

**Figure 12:**
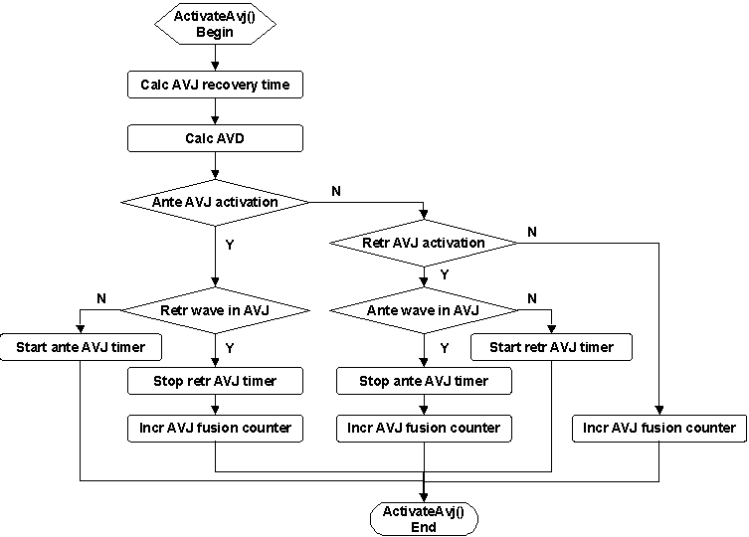
Flowchart of the *ActivateAvj *service called upon AVJ activation.

The AVJ is activated when V_m _≥ V_T_. The *ActivateAvj *service (Figure 12) calculates the AVD based on the measured AVJ recovery time. If the AVJ is antegrade excited, then an antegrade AVJ timer is started if there is no retrograde wave in the AVJ (expected normal condition); otherwise, the retrograde AVJ timer is stopped and the AVJ fusion counter is incremented (not applicable if the AVJ refractory period ≥ retrograde AVD). If the AVJ is retrograde activated, then a retrograde AVJ timer starts if there is no antegrade wave in the AVJ (expected normal condition), otherwise, the antegrade AVJ timer is disabled and the AVJ fusion counter is incremented (not applicable if the AVJ refractory period ≥ antegrade AVD). If the AVJ is simultaneously activated from both directions, an AVJ fusion is registered and no activation wave is generated.

**Figure 13:**
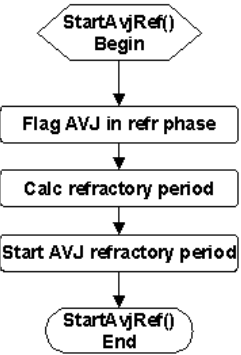
Flowchart of the *StartAvjRef *service called upon AVJ activation (to start the AVJ refractory period).

The activation of AVJ also starts the AVJ refractory period. The *StartAvjRef *service (Figure 13) simply flags the AVJ refractory phase, calculates the refractory period based on the measured AVJ recovery time, and then starts the AVJ refractory timer.

**Figure 14:**
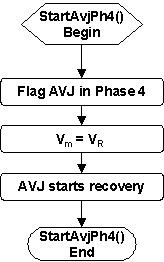
Flowchart of the *StartAvjPh4 *service called upon completion of the AVJ refractory period (to start the AVJ Phase 4).

The AVJ enters Phase 4 when the AVJ refractory timer reaches the end of the AVJ refractory period. The *StartAvjPh4 *service (Figure 14) then flags the AVJ in Phase 4, resets (V_m _= V_R_), and enables the AVJ to recover.

Of particular note is the fact that the above model framework allows different processes to generate the AF intervals, different protocols to generate the VP intervals, and different formulas to calculate the conduction time and refractory period (including the electrotonic modulation effect of the concealed conduction) of the AVJ.

## Results

**Figure 15:**
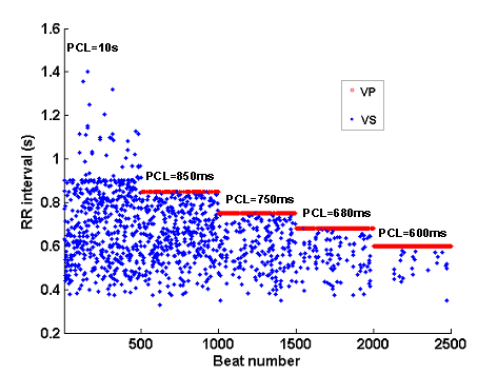
Example of simulated RR intervals in the presence of AF and VP.

In a typical application, Figure 15 shows the model-generated RR intervals in the presence of AF and VP. Five different sequences of 500 RR intervals were generated by varying the ventricular pacing cycle length (PCL). The arrival of AF impulses was modeled by a truncated Poisson process with a mean rate of 5/s [7]. Each AF impulse increments the V_m _by 15 mV, and the AVJ spontaneous Phase 4 depolarization rate is 30 mV/s. The ventricular response of intrinsic AF (PCL = 10 s) is characterized by short and irregular RR intervals. Progressively shortening the PCL (850 ms, 750 ms, 680 ms, 600 ms) results in increased percentage of VP (respectively 30%, 60%, 80%, 95%) and increased stabilization of the ventricular rate. In agreement with previous findings [12], VP not only eliminates long ventricular pauses, but also suppresses short intrinsic RR intervals in AF.

**Figure 16:**
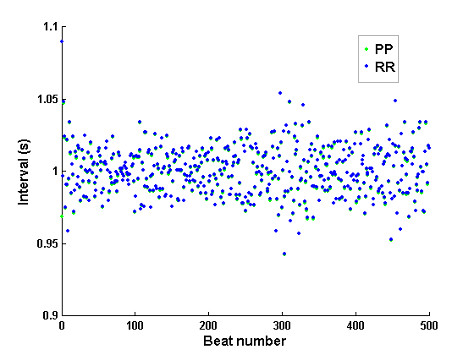
Example of simulated PP and RR intervals in sinus rhythm with heart rate variability.

Figure 16 illustrates a train of simulated 500 beat intervals (PP vs. RR intervals) in normal sinus rhythm. In this example, the random AF generator was replaced with a sinus rhythm generator according to a process described in [13]. Each of the atrial impulse (P wave) has supra-threshold strength, i.e., can bring V_m _directly to V_T_. The mean ± standard deviation (SD) of the atrial rate was set to 60 ± 1 bpm. The time series of PP intervals also has predefined heart rate variability that is characterized by a low frequency (LF) band centered at 0.1 Hz and a high frequency (HF) band centered at 0.25 Hz, and with LF/HF ratio of 0.5. Although the RR intervals generally match the corresponding PP intervals, slight difference between the two time series is apparent, which can be attributed to the rate-dependent variation of the AV conduction time.

**Figure 17:**
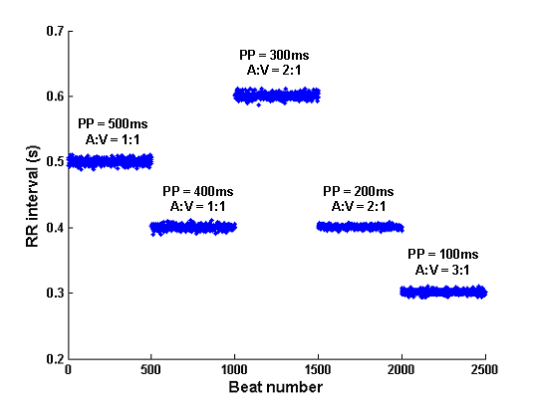
Example of simulated RR intervals in the presence of atrial flutter and AV block.

In another example, Figure 17 shows five runs of model-generated 500 beat RR intervals in the presence of typical atrial flutter and Mobitz type II AV block. In this case, the arrival of atrial impulses was simulated as a Gaussian process, and each atrial impulse has supra-threshold strength. From the first to the fifth sequence, the mean PP interval was decreased from 500 ms to 100 ms (with a step-size of 100 ms), all with a standard deviation (SD) of 10 ms. The AVJ refractory period, which is dependent on recovery time, was limited to a range between 250 ms and 500 ms. At a slower atrial rates (PP intervals of 400 ms and 500 ms), each atrial impulse is followed by a ventricular sense (known as 1:1 AV conduction). At higher atrial rates (PP intervals of 200 ms and 300 ms), every other atrial impulse is conducted to the ventricle (known as 2:1 AV conduction). For even higher atrial rates (a PP interval of 100 ms), 3:1 AV conduction occurs.

Details on how to generate above examples using the present AF-VP model are given in Appendix B.

## Discussion

The present AF-VP model described above can be easily extended to generate various types of ventricular rhythms by modifying the parameters specified in the model configuration file (see Appendices A, B).

From the system modeling perspective, the AVJ can be considered as a relay unit that connects its output (antegrade and retrograde activation waves) to the ventricle and atrium, which are respectively driven by two electrical sources, ventricular pacing and atrial impulse. Therefore, although the present model was described in the framework of atrial fibrillation and ventricular pacing, its usage is not limited to AF and VP only. Further applications of the model may include, but are not limited to:

- The AF generator can be modified to generate other random or deterministic processes, to simulate other types of atrial rhythms, including sinus rhythm with heart rate variability and atrial ectopic beats, paroxysmal or persistent atrial tachycardia, and programmed atrial pacing protocols [14].

- The AVJ properties (conduction timer and refractory period) can be modified to simulate various degrees of AV block including the second-degree Mobitz type I AV block (Wenckebach phenomena) [14] and the Mobitz type II block (Figure 17), or the uni-directional AV block. Alternatively, AV junctional tachycardia can be simulated by increasing the spontaneous Phase 4 activity of the AVJ [11].

- Various VP schemes can be tested by programming dynamic (instead of fixed) PCL. For example, different VP protocols can be implemented by continuously adjusting the PCL based on measured RR intervals, for the purpose of ventricular rate stabilization (VRS) in AF [15,16]. Two such algorithms, adaptive-VRS [16] and dynamic overdrive pacing [17], were included in the software as built-in functions for users' reference. In another example, the ventricular conduction time can be adjusted to simulate ventricle apical pacing or His bundle pacing, and quantitatively compare their VRS effects [18]. Moreover, the VP can be replaced by spontaneous ventricular activation to simulate the ventricular ectopic beats or episodes of ventricular tachycardia.

- The simulation output of the present model actually includes three closely coupled time series: PP intervals, RR intervals, and PR intervals (AV delays). By simulating various cardiac rhythms with different model parameter settings, these generated time series can be used to build a standard test platform for quantitative evaluation or comparison of different signal processing techniques, for example, the assessment of heart rate variability, the quantification of rhythm complexity, the classification of ECG rhythm types, and so on.

## Conclusion

We present the structure and implementation of a novel computer model, which is capable for synthesizing realistic time series of RR intervals under physiologic and pathologic conditions. The availability of this open source model promises to aid and stimulate future research in basic and applied cardiac electrophysiology involving RR interval analysis. Code to run this model, written in C, is available from PhysioNet [8].

## Competing interests

The author(s) declare that they have no competing interests.

## Authors' contributions

JL conceived, designed and implemented the computer model, and drafted the manuscript. DM and VL supervised the project, contributed to the discussion and interpretation of the results, and participated in manuscript revisions. GDC helped format the code and write the manuscript for open source release. All authors read and approved the final manuscript.

## Appendix A

The software reads the AF-VP model parameters from an external configuration file (default filename: config.txt), which can be modified by any text editor. Each model parameter is defined in a text line in the format of:

*parameter name *= *parameter value*

Any empty line is ignored. Besides, a line starting with '%', and the text following double slash '//' are considered as comments.

The model parameters are grouped into five parts, corresponding to the simulation environment and the four model components: the AF generator, the AVJ, the ventricle, and the right ventricular (RV) electrode (see [7] for detailed descriptions of the model parameters).

A sample configuration file, with suggested default model parameters and some comments, is listed below.

### % [Simulation environment]

fnRR = outrr1.txt // output RR interval filename

fnAA = outaa1.txt // output AA interval filename

fnAV = outav1.txt // output AVJ status filename

fnLOG = outlog1.txt // output event log filename

MAX_RR = 500 // max RR cycles to run for one simulation

MAX_TIME(s) = 1000.0 // max time to run for one simulation

Ts(s) = 0.001 // sampling interval (1000 Hz)

RR0(s) = 1.000 // initial RR interval

### % [Atrium model]

AA_MODEL = 0 // AA interval generator method

lambda(1/s) = 5 // mean rate of AF bombardment on AV junction

AAstd(s) = 0.0 // standard deviation of AA interval

dVmean(mV) = 15 // mean potential increment (dV) by AF bombardment

dVstd(mV) = 0 // standard deviation of dV

AtrDly(ms) = 0.03 // atrial conduction delay from AF source to AVJ

S1S2(s) = 0.2 // S1S2 interval of atrial pacing protocol (used in [12])

S2S3(s) = 0.5 // S2S3 interval of atrial pacing protocol (used in [12])

% AA model: 0-exponential, 1-inv poisson (n/a), 2-uniform, 3-Gaussion, % 4-S1S2 pacing, 5-S1S2/S2S3 pacing, 6-fixed

### % [AVJ model]

Vt(mV) = -40 // AVJ depolarization threshold potential

Vr(mV) = -90 // AVJ resting potential

dVdt(mV/s) = 33 // phase 4 depolarization rate

MinAVDa(s) = 0.070 // minimum anterograde AV conduction delay

MinAVDr(s) = 0.070 // minimum retrograde AV conduction delay

alpha(s) = 0.130 // max extension of the AV conduction time

tau_c(s) = 0.100 // time constant for AV conduction curve

MinRef(s) = 0.050 // minimum AVJ refractory period

beta(s) = 0.250 // max extension of the AVJ refractory period

tau_r(s) = 0.500 // time constant for AVJ recovery curve

Ref_std(s) = 0.000 // standard deviation of AVJ refractory period

delta = 10 // electrotonic modulation control (strength)

theta = 10 // electrotonic modulation control (time)

### % [Ventricle model]

AntDly(s) = 0.050 // antegrade conduction delay from AVJ to RV electrode

RetDly(s) = 0.150 // retrograde conduction delay from RV electrode to AVJ

ref(s) = 0.100 // ventricular refractory period

### % [RV electrode model]

VP_MODEL = 0 // ventricular pacing method

BI(s) = 0.80 // standby ventricular pacing basic interval

% VP_MODEL: 0-VVI, 1-adaptive VRS, 2-wittkampf, >2(default): VVI

## Appendix B

Various patterns of RR intervals can be generated using the present AF-VP model by modifying the configuration file described in Appendix A.

For example, Figure 15 (AF rhythm and VP) is produced by simply replacing the default values of the following two parameters as:

dVdt(mV/s) = 30

BI(s) = 10 // also 0.85, 0.75, 0.68, 0.60

Similarly, Figure 17 (atrial flutter rhythm) is generated by changing the default values of the following parameters to:

AA_MODEL = 3

lambda(1/s) = 2 // also 2.5, 3.333, 5, 10

AAstd(s) = 0.01

dVmean(mV) = 50

dVdt(mV/s) = 50

MinRef(s) = 0.250

BI(s) = 10

As laid out in Figs 3, 4, 5, 6, 7, 8, 9, 10, 11, 12, 13, 14, the software architecture of the model is constructed in a modular manner, to facilitate code change for achieving a particular aim. For example, in Figure 16 (sinus rhythm with normal heart rate variability), the PP intervals were generated using another open source model described in [13], instead of using any built-in AF interval generators (AA_MODEL). To achieve this, only two minor modifications are needed: (1) the PP intervals are imported from an external file (generated by the other model) during the 'Model Initialization' step (Figure 3); (2) the imported PP intervals are indexed to 'obtain the next AF interval' (Figures 6, 11), regardless of the parameter AA_MODEL setting.

## Supplementary Material

Additional File 1 Open-source of the AF-VP model. This file contains the complete archive of the AF-VP model source code. Click here for file
